# RNAseq Analysis of Brain Aging in Wild Specimens of Short-Lived Turquoise Killifish: Commonalities and Differences With Aging Under Laboratory Conditions

**DOI:** 10.1093/molbev/msac219

**Published:** 2022-11-01

**Authors:** Mariateresa Mazzetto, Cinzia Caterino, Marco Groth, Elisa Ferrari, Martin Reichard, Mario Baumgart, Alessandro Cellerino

**Affiliations:** Biology of Ageing, Leibniz Institute for Age Research—Fritz Lipmann Institute e.V. (FLI), Beutenbergstr. 11, 07745 Jena, Germany; Bio@SNS, Scuola Normale Superiore, Department of Neurosciences, Piazza dei Cavalieri 7, 56126 Pisa, Italy; Biology of Ageing, Leibniz Institute for Age Research—Fritz Lipmann Institute e.V. (FLI), Beutenbergstr. 11, 07745 Jena, Germany; Biology of Ageing, Leibniz Institute for Age Research—Fritz Lipmann Institute e.V. (FLI), Beutenbergstr. 11, 07745 Jena, Germany; Bio@SNS, Scuola Normale Superiore, Department of Neurosciences, Piazza dei Cavalieri 7, 56126 Pisa, Italy; Institute of Vertebrate Biology, Czech Academy of Sciences, Květná 8, 603 65 Brno, Czech Republic; Department of Ecology and Vertebrate Zoology, University of Łódź, 90-237 Łódź, Poland; Department of Botany and Zoology, Faculty of Science, Masaryk University, 611 37 Brno, Czech Republic; Biology of Ageing, Leibniz Institute for Age Research—Fritz Lipmann Institute e.V. (FLI), Beutenbergstr. 11, 07745 Jena, Germany; Biology of Ageing, Leibniz Institute for Age Research—Fritz Lipmann Institute e.V. (FLI), Beutenbergstr. 11, 07745 Jena, Germany; Bio@SNS, Scuola Normale Superiore, Department of Neurosciences, Piazza dei Cavalieri 7, 56126 Pisa, Italy

**Keywords:** killifish, *Nothobranchius furzeri*, brain aging, RNAseq, gene expression

## Abstract

A vast body of studies is available that describe age-dependent gene expression in relation to aging in a number of different model species. These data were obtained from animals kept in conditions with reduced environmental challenges, abundant food, and deprivation of natural sensory stimulation.

Here, we compared wild- and captive aging in the short-lived turquoise killifish (*Nothobranchius furzeri*). These fish inhabit temporary ponds in the African savannah. When the ponds are flooded, eggs hatch synchronously, enabling a precise timing of their individual and population age. We collected the brains of wild fish of different ages and quantified the global age-dependent regulation of transcripts using RNAseq. A major difference between captive and wild populations is that wild populations had unlimited access to food and hence grew to larger sizes and reached asymptotic size more rapidly, enabling the analysis of age-dependent gene expression without the confounding effect of adult brain growth.

We found that the majority of differentially expressed genes show the same direction of regulation in wild and captive populations. However, a number of genes were regulated in opposite direction. Genes downregulated in the wild and upregulated in captivity were enriched for terms related to neuronal communication. Genes upregulated in the wild and downregulated in captive conditions were enriched in terms related to DNA replication.

Finally, the rate of age-dependent gene regulation was higher in wild animals, suggesting a phenomenon of accelerated aging.

## Introduction

Aging is characterized as an age-dependent decrease in organismal fitness that results in increased mortality risk. One important source of aging is the accumulation of molecular damage that progressively impairs cellular homeostasis. High-throughput expression profiling techniques such as RNAseq provide a global and unbiased assessment of molecular changes associated with biological phenomena and are widely applied in biomedical and ecological research. A vast body of studies is available that describes age-dependent gene expression in relation to aging in a number of different model species (Zahn et al. 2006; Aramillo Irizar et al. 2018; Schaum et al. 2020; Aging Atlas Consortium 2021). Yet, most data are derived from animals kept in highly controlled laboratory conditions with reduced environmental challenges and pathogen load and with a considerably modified diet compared with natural nutrition (Zak et al. 2022). Captive animals, on the other hand, may be deprived of natural sensory stimulation and opportunities for physical activities with negative consequences on brain development and functions (van Praag et al. 2000; Sale et al. 2014; Pedersen 2019) and—on the other hand—have a supply of energetically rich diet supporting faster growth. To what extent the differentially expressed genes and pathways identified under artificial laboratory conditions reflect aging processes under natural conditions remains unclear.

Gene expression profiles associated with aging in natural conditions are available for wolves (Charruau et al. 2016) and bats (Huang et al. 2019) but—to our knowledge—not for model organisms such as rodents or fruit flies. In the present paper, we set out to compare age-dependent gene expression during adult life in a vertebrate model species, the annual fish *Nothobranchius furzeri*. *Nothobranchius furzeri* is a small (6 cm) annual fish that inhabits temporary ponds in Southeastern Africa subject to the monsoonal seasonality. The duration of these habitats usually varies from 1 to 4 months after which all fish die. However, fish density strongly declines, and all fish often disappear, long before desiccation (Vrtilek, Zak, Polacik et al. 2018). The lifespan of wild-derived *N. furzeri* strains raised in captivity is limited to 7–8 months (Terzibasi et al. 2008). This species has become a new model organism since a compressed adult lifespan is associated with rapid physiological decay. A large number of typical vertebrate aging phenotypes at the neuro-muscular, histological, and cellular/molecular levels are described (for systematic reviews, see Cellerino et al. 2016; Platzer and Englert 2016; Hu and Brunet 2018; Poeschla and Valenzano 2020). Examples are reduced locomotor activity and impairment in learning paradigms (Valenzano et al. 2006), accumulation of the fluorescent age pigment lipofuscin (Terzibasi et al. 2008), apoptosis (Di Cicco et al. 2011), telomere erosion (Hartmann et al. 2009), and reduced mitochondrial function (Hartmann et al. 2011). RNAseq was applied to describe the global, age-dependent regulation of transcripts in different organs (Baumgart et al. 2014, 2016). The comparison of these datasets with similar datasets obtained in zebrafish, mouse, and humans revealed a consensus vertebrate transcriptional pattern that is conserved across species (Aramillo Irizar et al. 2018).

A large amount of information is available concerning *N. furzeri* ecology (reviewed in the study by Reichard and Polacik 2019), including detailed data on demographic parameters from wild populations. These studies demonstrated that the growth rate of *N. furzeri* in natural habitats is dependent on population density (Vrtilek et al. 2019) and is faster than in captivity, with animals in low-density habitats able to reach sexual maturity in 12 days (Vrtilek, Zak, Psenicka et al. 2018). *Nothobranchius furzeri* therefore represents an ideal species to compare age-dependent gene expression in captivity and in natural conditions.

For this study, we selected the population A41 (termed as Ch1 in [Vrtilek, Zak, Polacik et al. 2018]), which belongs to the Chefu phylogeographic lineage that comprises also the MZM-0410 captive strain (originally collected from a pond only 21 km apart) for which datasets of age-dependent genome-wide transcriptome regulation are available. In 2016, wild individuals of *N. furzeri* from population A41 were collected over the entire season, and their birth date was estimated from daily otolith increments (Vrtilek, Zak, Polacik et al. 2018). The collection of fish tissues from individuals of specified ages was completed in the field to be compared with captive animals of similar age points. An important characteristic of this wild population is that no growth was observed during the observation period (sample collection started at age of 39 days, when body size reached the asymptote; supplementary fig. S1, Supplementary Material online). Therefore, age-dependent expression was not confounded with growth-dependent expression. We focused on gene expression in the brain because cellular composition in the *N. furzeri* brain does not change during aging (Kelmer Sacramento et al. 2020) and the brain tissue is expected to respond strongly to environmental stimuli. Since studies of sex-dependent gene expression are lacking for *N. furzeri*, we also took the opportunity to compare age-dependent gene expression in male and female individuals.

## Results

### Effects of Age and Sex on Brain Gene Expression in the Wild

**Fig. 1.**
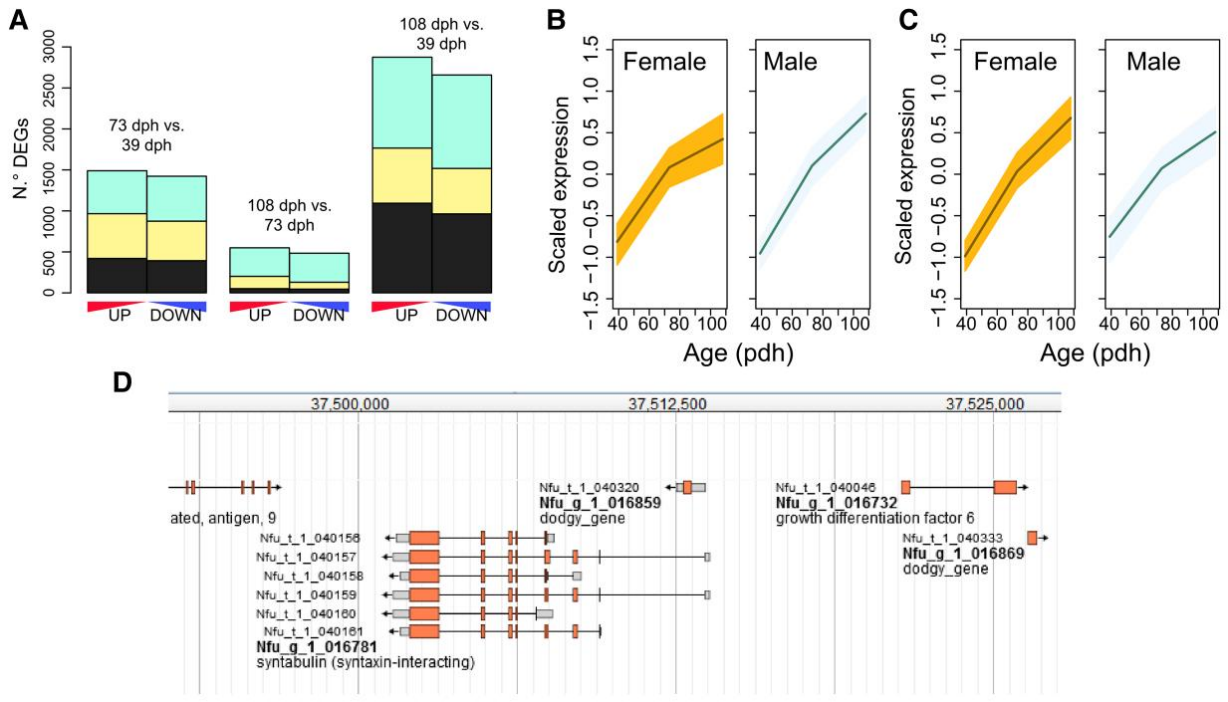
Sex differences in gene expression. (*A*) Number of DEGs for the different aging comparisons (73 vs. 39 dph, 108 vs. 73 dph and 108 vs. 39 dph) in female and male samples. The number of DEGs was computed after performing differential gene expression analysis with the DESeq2() package and filtering for *P*adj < 0.05. Pale gray represents DEGs detected in females only, purple DEGs detected in males only and dark gray DEGs detected in both sexes. (*B* and *C*) Transcriptional aging biomarkers in female (*B*) and male (*C*) brain samples. Genes with positive monotonic expression profile among the time points were computed with the Cuzick method for each sex, filtered for *P* < 1e−04 and z-normalized. Points represent means and shadowed areas intervals of confidence; normalized expression for the same genes are plotted in the other sex for comparison. (*D*) Visualization of the genomic neighborhood of the syntabullin (SYNTB) gene from *N. furzeri* Genome Browser NFINgb (https://nfingb.leibniz-fli.de/). Numbers on the top bar represent positions on the synteny group 5. SYNTB is one of the sex-dependent DEGs found in wild samples as is the nearest-neighbor of GDF6, the sex-determining locus in *N. furzeri* (Reichwald et al. 2015).

We sequenced RNA extracted from the brains of wild *N. furzeri* collected at 3 time points (39, 73, and 108 dph) from both sexes for a total of 22 samples. These ages are similar to the age of 35 and 94 dph that were previously studied in captive populations (Baumgart et al. 2014, 2016), so we can compare gene expression in animals of similar chronological age. In captive animals, these ages correspond to the life stages of young fish soon after sexual maturation and young adulthood. Due to the more rapid growth and sexual maturation in wild conditions, similar chronological ages do not imply similar “biological” ages or life stages. We computed differentially expressed genes (DEGs) in all the pairwise comparisons (73 vs. 39 dph, 108 vs. 73 dph, and 108 vs. 39 dph) for each sex separately, and we detected no major sex-related differences in the number of DEGs (fig. 1*A* and supplementary table S1, Supplementary Material online). We then computed putative aging biomarkers defined as upregulated genes with a monotonic trend for each sex independently. Markers detected in one sex had very similar expression profiles in the opposite sex (fig. 1*B* and supplementary table S2, Supplementary Material online), demonstrating that sex has little influence on the genome-wide regulation of transcripts by age in the brains of wild animals.

A generalized linear model, as implemented in DESeq2 (Love et al. 2014), was applied to separate the effects of sex and age on gene expression. Only 36 sex-dependent DEGs were detected (supplementary table S3, Supplementary Material online). Significantly regulated genes such as SYBU (syntabullin), which is the nearest-neighbor gene of GDF6 (growth differentiation factor 6), the sex-determining locus of *N. furzeri* (Reichwald et al. 2015) (fig. 1*C*), and Cytochrome P450 Family 19 Subfamily A Member 1, a gene that catalyzes the formation of aromatic C18 estrogens from C19 androgens (Corbin et al. 1988; Baravalle et al. 2017) are related to mechanisms of sex differentiation.

**Fig. 2.**
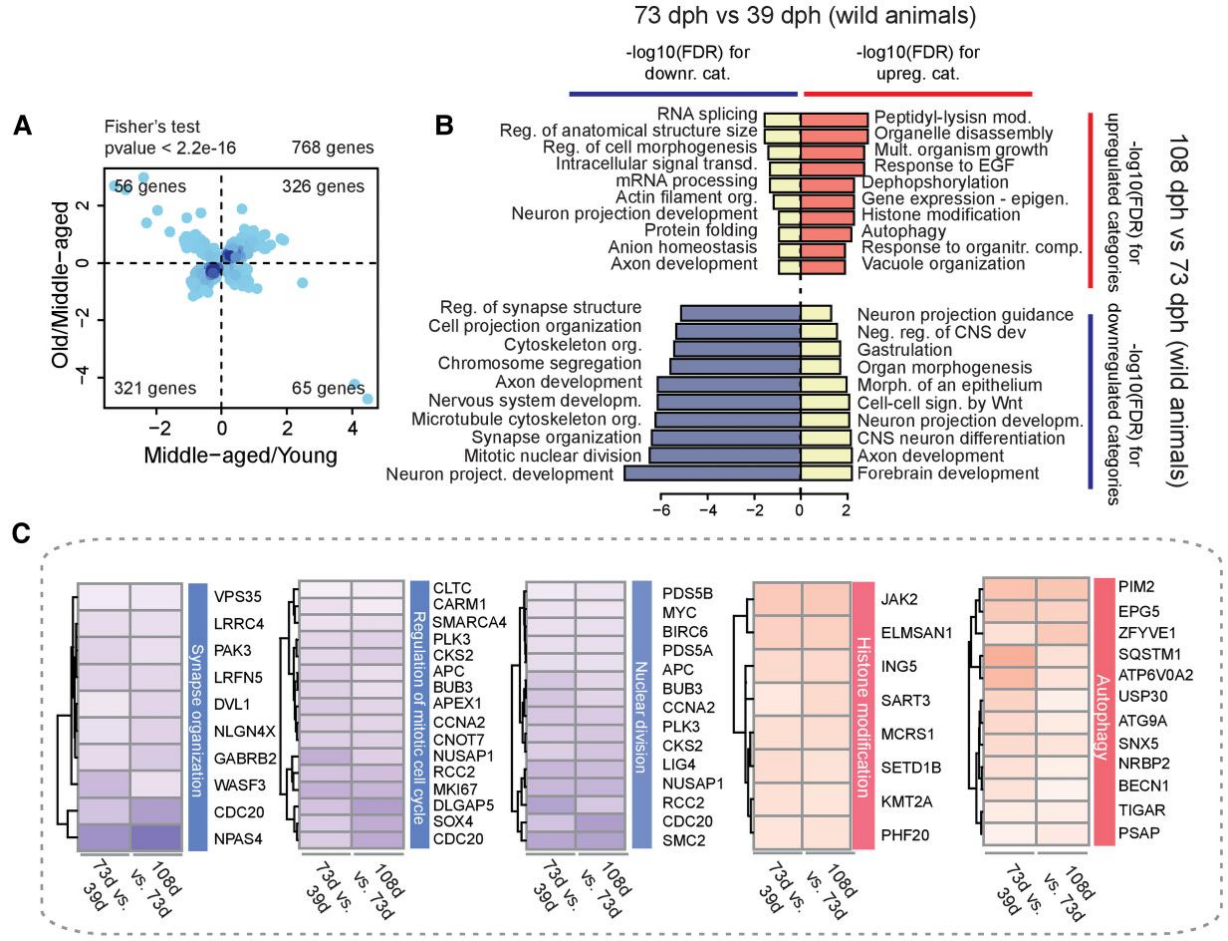
Aging-dependent pathways in wild populations. (*A*) Scatter plots of aging-dependent fold-changes in wild populations: 73 wph versus 39 dph comparison is shown on the *x*-axis and 108 versus 73 dph comparison on the *y*-axis. DEGs for both the comparisons are plotted after filtering for *P*adj < 0.05. The density of genes is coded by the intensity of the blue hues, and the number of genes in each quadrant is reported in the respective vertex. (*B*) GO biological process overrepresentation analysis of the genes contained in the four quadrants of (*A*). Length of the bars represents significance as −log_10_ of the FDR. (*C*) Heatmaps of (from left to right) synapse organization, regulation of mitotic cell cycle, nuclear division, histone modification, and autophagy; genes significantly affected for both the comparisons are displayed. Upregulated genes (with logFC > 0) are colored as red boxes while downregulated genes (with logFC < 0) are displayed as blue boxes.

Two aging-related contrasts were performed in the wild animals: for each sex separately and combining male and female samples. We analyzed genes expressed in the brain that were detected as differentially expressed both during early- (73 vs. 39 dph) and late-adulthood (108 vs. 73 dph) and plotted the log_2_ (fold change) for the first contrast on the *X*-axis and the log_2_ (fold change) for the second contrast on the *Y*-axis. Genes DE in both comparisons showed a positive correlation in their fold-changes, and the data points were more concentrated in quadrants I and III (i.e., genes with either negative or positive monotonic trend, Fisher’s exact test: *P* < 2.2e−16) (fig. 2*A*, supplementary table S4, Supplementary Material online), indicating a progressive nature of age-dependent expression changes.

In order to identify the biological processes that are most affected during aging of wild fish, we performed Gene Ontology (GO) overrepresentation analysis for the genes in the four quadrants of figure 2*A*. Results are reported in figure 2*B* and supplementary table S5, Supplementary Material online. Genes with monotonic downregulation showed an overrepresentation of categories related to cell cycle, mitotic nuclear division and synapse organization, and axonogenesis, indicating a progressive age-dependent decrease in mitotic activity (neurogenesis) and formation of neural connections (fig. 2*B* and *C*). Genes with monotonic upregulation showed overrepresentation of categories related to peptidyl lysine modifications, epigenetics, and autophagy (fig. 2*B* and *C*). Genes with a reversal in their regulation also showed overrepresentation of specific categories: genes initially downregulated for mRNA processing, RNA splicing, and protein folding, and genes initially upregulated for central nervous system (CNS) development (fig. 2*B*).

### Wild and Captive Animals Differ in the Age-Dependent Regulation of Genes Related to DNA Repair and Neural Functions

**Fig. 3.**
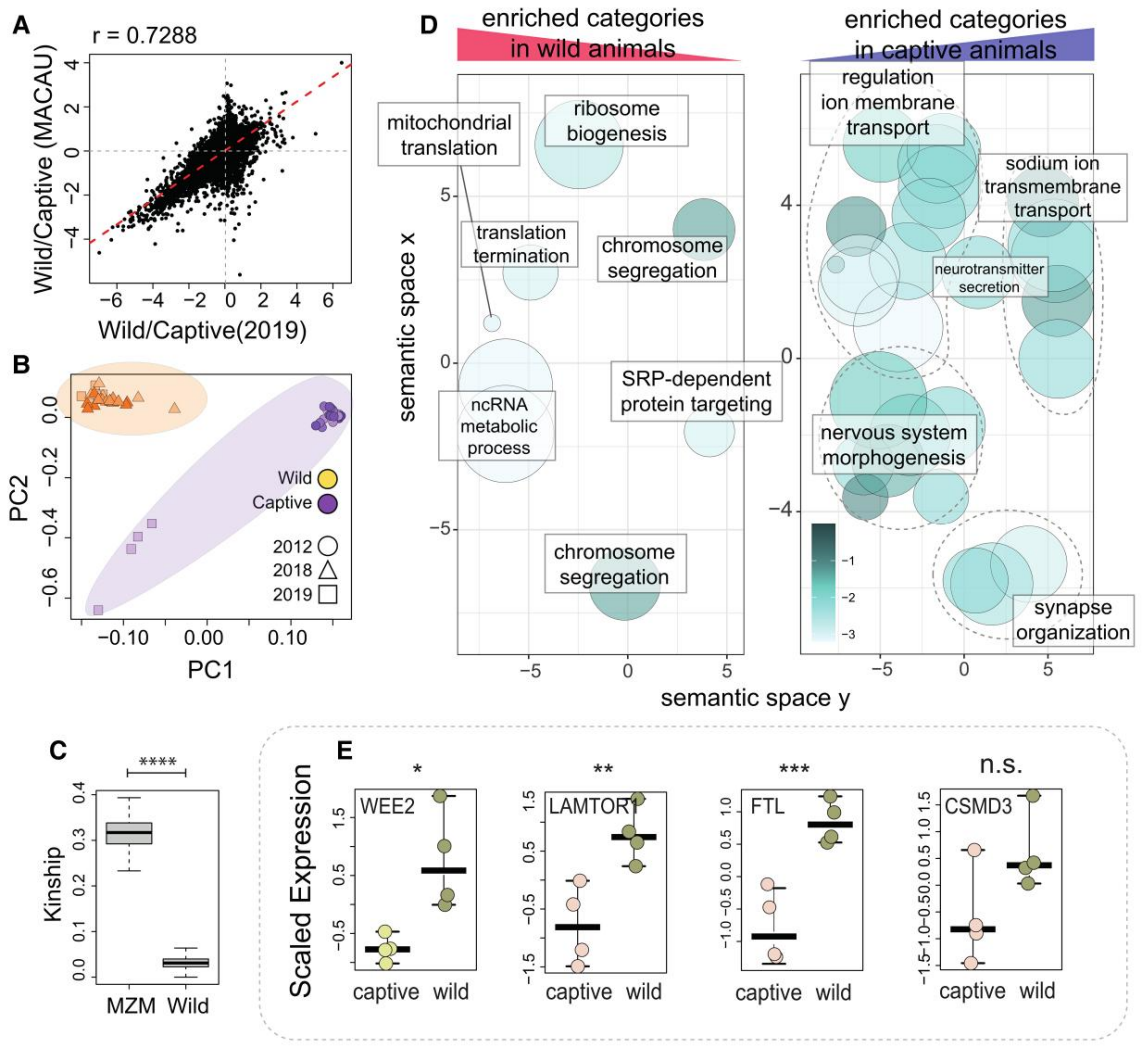
Comparison of strain-dependent gene expression between wild and captive populations. (*A*) Scatter plots of condition effect computed by MACAU on the complete dataset on the *X*-axis with the fold-changes detected by DESeq comparing one dataset of wild versus captive (MZM-0410) young samples (39 dph for wild samples, 5 wph for MZM-0410 samples, all sequenced in a single batch) on the *Y*-axis. Both correlation coefficients and the linear regression line are displayed. (*B*) PCA plot of genetic relatedness between the samples based on IBD. The shape of the points indicates the sequencing batch, and the color distinguishes captive and wild specimens. (*C*) Boxplots of pairwise kinship in the wild and captive samples (*D*) Gene enrichment results for condition-dependent transcripts (wild vs. captive). Enrichment analysis was performed by applying the gage package (Luo et al. 2009) to the results of each analysis corresponding to the two axis of (*A*) separately, and only categories significantly modulated in both RNAseq experiments were further clustered based on their semantic similarity using REVIGO (Supek et al. 2011). Significance of the single categories is displayed by color code; one single representative GO term was use to annotate clustered terms (light blue dashed lines). (*E*) Experimental validation of differential expression for selected genes: from left to right, WEE2, FTL, LAMTOR1, and CSMD3 are displayed as top DEGs between wild and captive populations. For the evaluation of their expression, RT-qPCR was performed using female and male samples at 39 dph for wild samples and 5 wph for MZM-0410 samples. Each point represents a replicate, and the middle segment represents the median of the distribution. *T*-test was used to assess statistical significance. *P*-values legend used in the figure: *<0.05, **<0.01, and ***<0.001.

We set to detect the effects of captive versus wild condition on gene expression. We performed a second experiment where we compared four 34–37-day-old captive male fish and four 39-day-old wild male fish processed in parallel to eliminate batch effects linked to the RNAseq process, and we also compared the result of our first RNAseq experiment with a publicly available RNAseq dataset of age-dependent gene expression comprising five time points that partially overlap with those studied in the wild (Baumgart et al. 2016) (fig. 3*A* and supplementary table S6, Supplementary Material online).

Since the genetic structure of these populations is not known, we first computed identity by descent by analyzing SNPs in the RNA sequence data using the package SNPRelate (Zheng et al. 2012). This analysis showed that the wild population is genetically distinct from the captive MZM0410 strain (fig. 3*B*). In addition, the two captive samples sequenced in 2012 and 2019 were also genetically distinct, indicating a drift in this population. Analysis of kinship and diversity indicated that the captive population has on average a lower diversity (measured as the distance from the centroid) and a higher average kinship (fig. 3*C*).

To correct for the effect of genetic relatedness on gene expression, we used MACAU, an approach based on Poisson mixed models that is specifically designed to correct for the effects of genetic relatedness on gene expression by modeling the heritability of expression levels (Sun et al. 2017).

We first created a model where the condition is the independent variable and age and batch are treated as covariates. The analysis with MACAU resulted in 11,379 DEGs (false discover rate, FDR < 0.1) (supplementary table S7, Supplementary Material online) between wild and captive animals. As this number is very high and corresponds to roughly 40% of the genes in the genome, we compared these results with those obtained by applying DESeq2 only to the dataset of four wild and four captive fish processed in parallel. The number of DEGs with *P*adj < 0.05 was 8,115. Therefore, the number of DEGs we obtain with MACAU is not unreasonable and probably reflects the pervasive effect of the condition on gene expression. Moreover, we correlated the fold-changes measured with DESeq2 with the slopes calculated with MACAU on the complete dataset (fig. 3*A*, supplementary table S6, Supplementary Material online) and detected a high degree of correlation.

Generally applicable gene set enrichment Generally Applicable Gene-set (GAGE) (Luo et al. 2009) was applied in order to detect pathways whose expression is condition-dependent between wild and captive animals. In order to increase the stringency of the GAGE analysis, we performed GAGE analysis also on the DESeq2 results that we obtained by analyzing a single batch. We then retained as significant only the terms that were detected in both analyses. As the number of significantly overrepresented terms (FDR < 0.1) is large, we used REVIGO (Supek et al. 2011) to cluster GO categories based on semantic similarities (fig. 3*D*). Genes upregulated in wild animals showed enrichment of categories related to mitochondrial biogenesis and energetics, RNA processing, and to mitosis. On the other hand, genes upregulated in captive animals were enriched in categories related to development, behavior, and function of the nervous system as well as ion transport (supplementary table S8, Supplementary Material online). Among the top differentially regulated genes, we found WEE2, a protein kinase that phosphorylates inhibitory sites in CDK1 (Leise and Mueller 2002), FTL, the ferritin light chain, LAMTOR1, an anchor protein that creates an interface for the mTORC1 complex on late endosomes thereby regulating cell metabolism (Bar-Peled et al. 2012) and CSMD3, a gene coding for a postsynaptic protein highly expressed in the brain that regulates dendrite development (Mizukami et al. 2016) and neuronal maturation (Gutierrez et al. 2019). Differential expression of these genes was validated by quantitative PCR (fig. 3*E*).

**Fig. 4.**
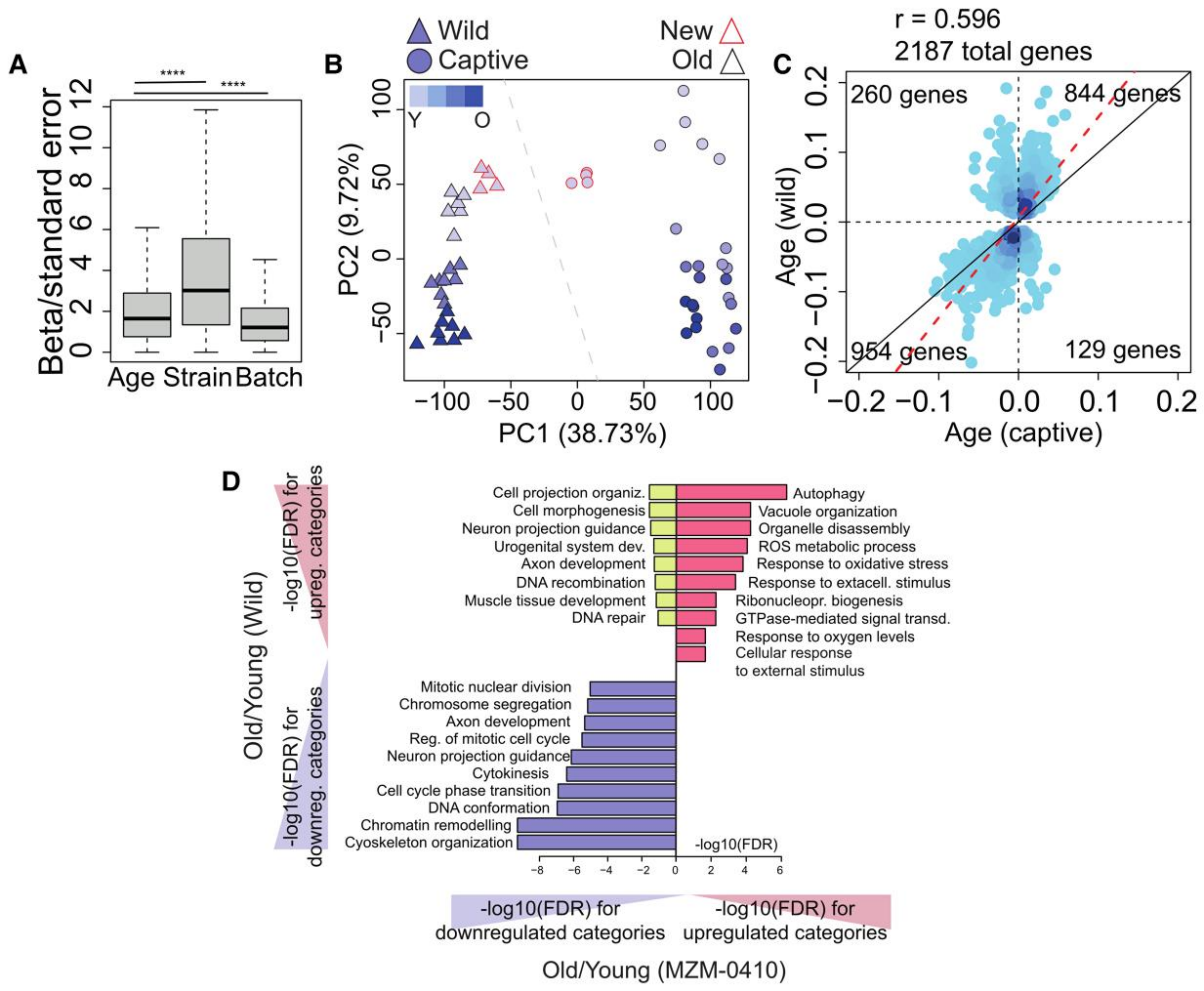
Comparison of gene expression during aging in the wild and in captivity. (*A*) Boxplot of the transcriptome-wide distribution of the absolute weight of the different variables on gene expression as implemented by MACAU in a Poisson mixed model. *T*-test was used to assess statistical significance. *P*-values legend used in the figure ***<0.001, ***<0.0001. (*B*) Visualization by PCA of the sample relationships based on transcriptome. In the PCA plot, different strains/populations are coded by shape (triangles for wild, and circles for MZM-0410) and progression from early to older time points is displayed as increasing saturation. Time points are 39, 73, and 108 dph for the wild samples and 5, 12, 20, 27, and 39 wph for the captive samples. The red outline identifies samples from the 2019 sequencing batch. (*C*) Comparison between age-dependent effects of gene expression, quantified as the β coefficient in the Poisson mixed model, in the brain of captive MZM strain (*x*-axis) and wild (*y*-axis) *N. furzeri*. Only genes significantly regulated with *P*adj < 0.05 in both contrasts are shown. Pearson correlation and total number of genes, as well as number of genes per quadrant are shown. The density of genes is coded by the intensity of the blue hues. The red line represents the best fit of linear regression between the two variables, and the black line represents a line of intercept = 0 and slope = 1. (*D*) GO biological process overrepresentation analysis of the genes contained in the four quadrants of (*C*). Length of the bars represents significance as −log_10_ of the FDR.

We created two further models—one for captive fish and one for wild fish. In these models, age is the independent variable and batch is treated as a covariate. This analysis revealed that the effect of captive versus wild condition is larger than the effect of age and that the effect of batch is smaller than that of either of the two (fig. 4*A*). Principal component analysis (PCA) was applied to visualize relationships between the origin of the sample and the effects of age on global gene expression. As expected, the first component (69.52% of variance) separated fish conditions (wild vs. captive); the second component (5.46% of variance) identified an effect of age on global gene expression that was similar in the two conditions (fig. 4*B*). Notably, PC1 separated by condition also the samples of the new sequencing batch. However, the separation of these samples on PC1 was smaller than the samples of the 2012 and 2018 sequencing, indicating that batch effects contribute to sample separation, although to a lesser extent than captive- versus wild condition. This is consistent also with the distribution of effect sizes of condition, age, and batch as measured by MACAU and reported in figure 4*A*.

In order to compare aging-related differences in RNA expression between the two conditions, we then plotted the effect of age (i.e., the weight β of age in the Poisson mixed model) in the captive animals on the *X*-axis and in the wild animals on the *Y*-axis. The DEGs showed preferentially the same direction of regulation in the two conditions and were concentrated in quadrants I and III with a highly significant positive correlation (fig. 4*C*, supplementary table S9, Supplementary Material online). Notably, virtually all points in quadrant I are located above the diagonal of that quadrant, indicating that the age-dependent rate of upregulation is larger in wild fish. A similar, but smaller, effect can be noticed in quadrant III, where most points are located below the diagonal.

These results suggest a deceleration of transcriptional aging in captive animals (as shown in the next paragraph).

**Fig. 5.**
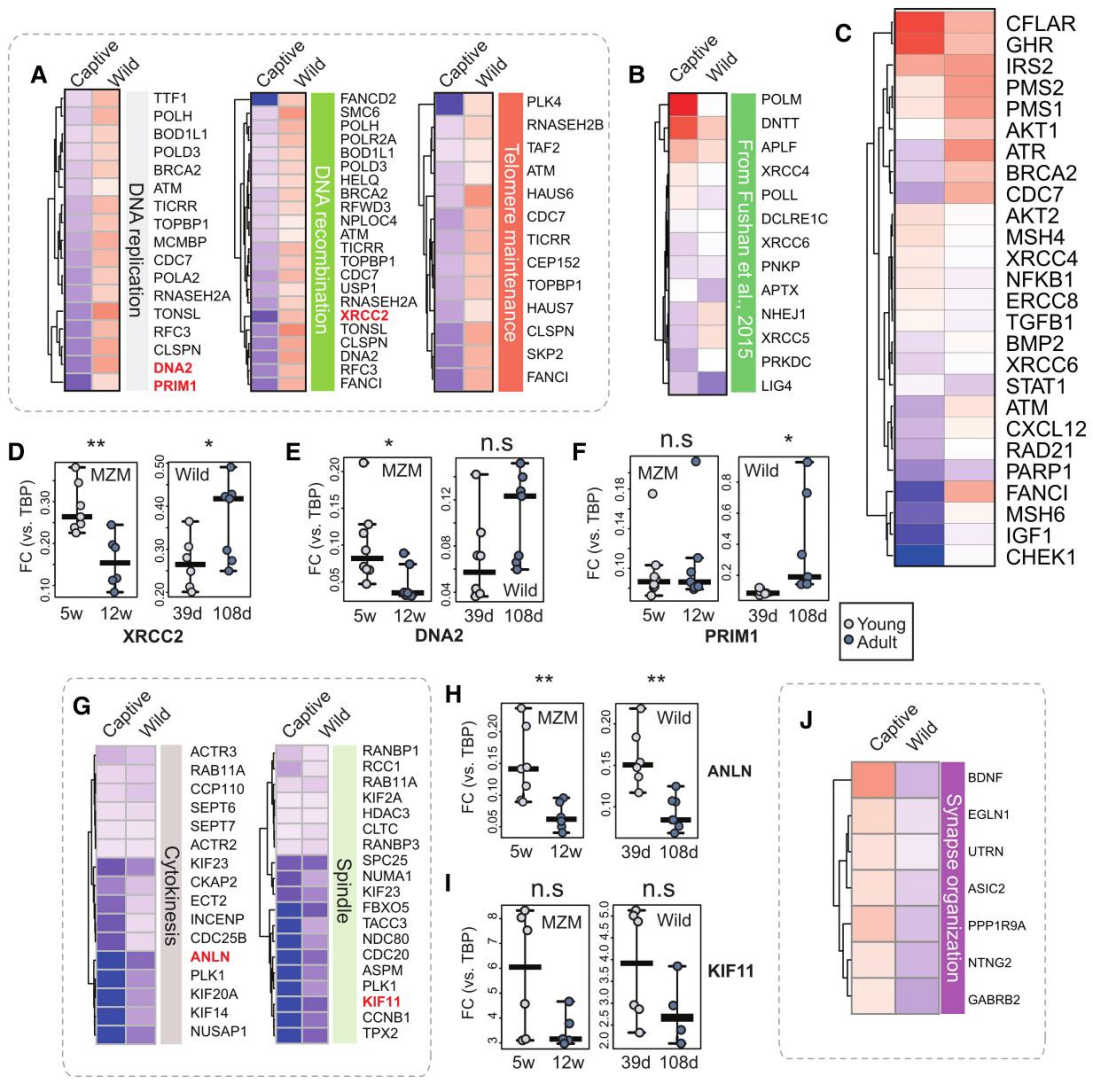
Discordant gene regulation in the wild and captivity. (*A*) Heatmaps of DEGs belonging to selected GO categories: only genes significantly downregulated in captive populations and significantly upregulated in wild populations are displayed. Upregulated genes (with logFC > 0) are colored as red boxes while downregulated genes (with logFC < 0) are displayed as blue boxes. The regulated genes are also compared with two sets of genes whose expression is positively correlated with lifespan in mammals. (*B*) Heatmap of DNA repair genes whose expression is positively correlated with lifespan taken from Fig. 6 of Fushan et al. (2015). (*C*) Heatmap of genes whose expression is positively correlated with lifespan from Lu et al. (2022). Genes selected for experimental validation are highlighted in red in (*A*): XRCC2 (*D*), DNA2 (*E*), and PRIM1 (*F*). For the evaluation of their expression, RT-qPCR was performed using female and male samples at different ages (39 and 108 dph for wild, 5 and 12 wph for captive, named “young” and “adult”). Each point represents a replicate, and the middle line represents the median. *T*-test was used to assess statistical significance. (*G*) Heatmaps of DEGs belonging to selected GO categories: only genes significantly downregulated both in captive- and wild samples are displayed. Genes selected for validation are highlighted in red: ANLN (*H*), and KIF11 (*I*). For the evaluation of their expression RT-qPCR was performed using female and male samples at different ages (39 and 108 dph for wild, 5 and 12 wph for captive, named “young” and “adult”). Each point represents a replicate, and the middle line represents the median. *T*-test was used for statistics. (*J*) Heatmap of DEGs belonging to the “Synapse organization” category that is significantly upregulated in captive- and significantly downregulated in wild samples.

We performed GO terms overrepresentation analysis for the genes in the four quadrants of figure 4*C*. Results are reported in figure 4*D* and supplementary table S9, Supplementary Material online. Genes downregulated in captivity, but upregulated in the wild during aging were enriched in terms related to DNA repair, cell cycle and synapse organization (figs. 4*D*, 5*A*, 5*D*–*F*, and 5*J*). Interestingly, the expression of genes related to DNA repair and non-homologous end joining was positively correlated with longevity in two different comparative RNAseq studies of mammals spanning a large spectrum of lifespans (Fushan et al. 2015; Lu et al. 2022). These same genes are frequently regulated in opposite directions in captive and wild animals (fig. 5*B* and *C*). We set to validate these differences in an independent biological sample using qPCR. Since it is not possible to directly compare the “biological” age in the two conditions, we decided to compare similar calendar ages, that is, 39 and 109 dph for the wild fish and 34–37 and 83–87 dph for the captive fish. Among the genes that are downregulated in captive animals but upregulated in wild animals, there were DNA2, a key enzyme involved in DNA replication and DNA repair in the nucleus and mitochondrion (Zheng et al. 2020), XRCC2 and RAD51, proteins involved in the homologous recombination DNA repair pathway (Suwaki et al. 2011), and PRIM1, a subunit of the DNA primase complex (Loeb and Monnat 2008). Divergent regulation of these genes was confirmed by qPCR (fig. 5*D*–*F* and supplementary table S10, Supplementary Material online).

Genes upregulated during aging in both wild and captive animals were most enriched in categories related to autophagy and response to reactive oxygen species (fig. 4*D*). Genes downregulated during aging in both conditions showed a highly significant enrichment for cytokinesis and mitotic nuclear division categories, chromatin remodeling, and nervous system development (figs. 4*D* and 5*E* and supplementary table S10, Supplementary Material online). As representative examples of concordant downregulation, we selected the genes ANLN, required for cytokinesis and essential for the structural integrity of the cleavage furrow (Kim et al. 2017), and KIF11, a motor protein required for establishing a bipolar spindle during mitosis (Johnson et al. 2014) (fig. 5*F* and *G* and supplementary table S10, Supplementary Material online).

### An “Accelerated” Aging Profile in Wild Animals

**Fig. 6.**
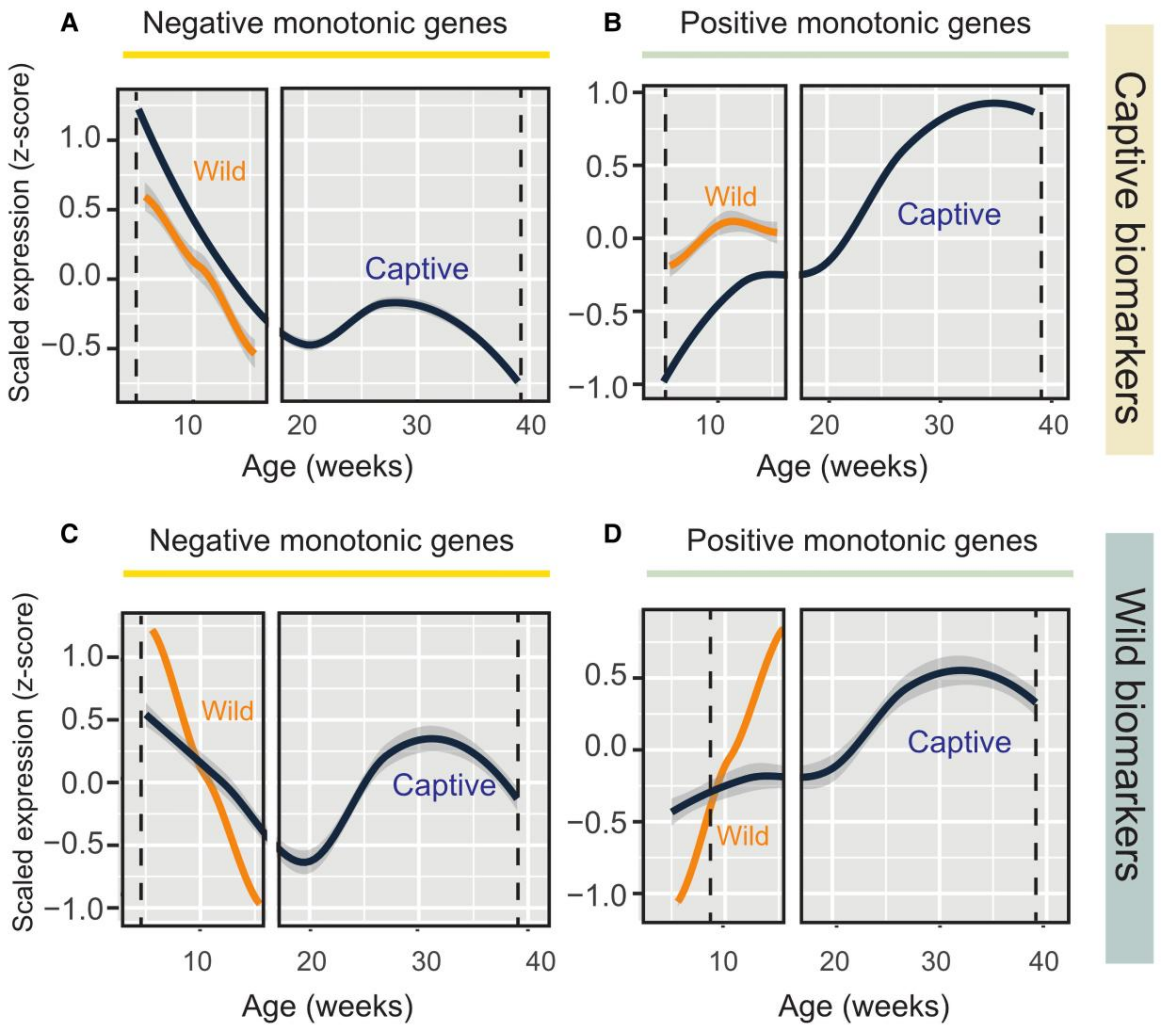
Transcriptional aging biomarkers. (*A* and *B*) Expression profile of aging biomarkers identified in captive samples in captive (blue) and wild (gold) animals: both downregulated (*A*) and upregulated (*B*) biomarkers are shown. The list of genes was taken from supplementary table S10, Supplementary Material online. (*C* and *D*) Expression profile of aging biomarkers identified in wild samples in captive (blue) and wild (gold) animals: both downregulated (*C*) and upregulated (*D*) biomarkers are shown. Scaled expression values are plotted with the regression line using ggplot2 package.

In order to quantify the differences in aging profile in the two conditions, we defined as transcriptional biomarkers of aging those transcripts with either negative or positive monotonic dependency on age in either captive- or wild fish (fig. 6*A*–*C* and supplementary table S11, Supplementary Material online). These transcripts showed the same direction of regulation in either condition but their age-dependency varied according to the condition. When captive biomarkers are used as a reference, wild- and captive fish appear to have similar slopes, but different intercepts, so that the curves of wild fish are left-shifted. When wild biomarkers are used as a reference, however, the slopes of age-dependent changes were clearly less steep in the captive fish, particularly for the genes upregulated with age. As mentioned earlier, a larger rate for age-dependent changes in wild fish can also be noted in the scatterplot in figure 4*C*.

These results strengthen the suggestion that captive animals show a “decelerated” aging profile when compared with the captive animals.

## Discussion

In this paper, we studied age-dependent gene expression in the brain of wild individuals of a *N. furzeri* population that was followed longitudinally over its natural lifespan (Vrtilek, Zak, Polacik et al. 2018). The time points analyzed correspond to adult stages, and it is of particular relevance that all fish in this study have reached asymptotic body size prior to the first sampling, and hence no further growth was observed across sampling age points. This enabled us to separate the effects of aging from the effects of growth that are confounded in the captive fish populations.

The first result of this study is that only minor sex differences were detected in brain gene expression and the pattern of age-dependent regulation. This is rather surprising given the marked sexual dimorphism of *N. furzeri* (Cellerino et al. 2016), the fact that sex-dependent changes in gene expression during aging were described in model organisms and humans (Yuan et al. 2012; Wruck and Adjaye 2020; Zhao et al. 2020) and that *N. furzeri* males are subject to a higher mortality in the wild (Vrtilek, Zak, Polacik et al. 2018). However, the captive population of *N. furzeri* do not show sex differences in the aging component of their survival or in expression of age-dependent markers, suggesting that the intrinsic rate of aging is not sex-dependent in this species (Reichard et al. 2022). Instead, increased male mortality is related to sex-specific predation and male–male competition for reproductive opportunities (Reichard et al. 2014). It should also be noted that analysis of zebrafish brains also revealed only ∼100 genes expressed in a sex-specific manner (Yuan et al. 2019), suggesting that transcriptional dimorphism may be less developed in teleost fishes.

The majority of the changes that were observed in the wild population were progressive; that is, the direction of the regulation was consistent in the two comparisons 39 versus 73 dph and 73 versus 108 dph. This indicates that condition-dependent mortality and the associated selection do not have a large impact on the data we obtained. Age-dependent downregulation affects primarily genes related to neurogenesis and synaptogenesis. Depression of adult neurogenesis is a typical age-related trait in vertebrates (Pekcec et al. 2008; Ben Abdallah et al. 2010; Tozzini et al. 2012; Edelmann et al. 2013), and downregulation of synaptic proteins is a typical feature of human brain aging (Lu et al. 2004; Somel et al. 2010; Berchtold et al. 2013; Wruck and Adjaye 2020). We show that these aspects of aging are conserved in the wild population. Upregulated genes showed overrepresentation of a number of different categories. Two particularly interesting terms were lysosome and epigenetic regulation of gene expression and histone modifications (supplementary table S5, Supplementary Material online). Upregulation of genes coding for lysosomal proteins is among the most conserved transcriptional signatures of aging across species (Zahn et al. 2006; de Magalhaes et al. 2009; Aramillo Irizar et al. 2018). Epigenetic regulation was described in captive *N. furzeri*. In particular, upregulation of repressive histone marks such as H3K27 trimethylation and downregulation of activating histone marks was detected (Baumgart et al. 2014; Cencioni et al. 2019).

Despite these conserved age-related patterns, baseline differences in expression between wild and captive populations were observed for a large number of genes.

The origin of this difference is likely manifold. Differences in nutrition almost certainly play an important role. First, captive animals are fed exclusively with *Chironomus* larvae. This diet does not correspond to the dietary protein target of wild fish (and is instead richer in lipids) and leads to lower body condition, overfeeding, and male liver enlargement (Zak et al. 2022). Second, wild animals have regular access to food over daytime rather than intermittent feeding once or twice a day. These two factors result in much faster growth of wild fish that, unlike captive animals, reach asymptotic size within 5 weeks. In wild fish, density-dependent effects may slow growth when resources become limited (Vrtilek et al. 2019), but this is not the case in our study population (A41). Indeed, the study population was specifically selected because it does not suffer from density-related restrictions, and this is further confirmed by the continuous presence of food in the gut throughout the day (Zak et al. 2019). A second highly relevant aspect of differences between wild and captive fish is thermal stress. Wild animals are subject to diel oscillations in temperature that reach almost 20 °C (Zak and Reichard 2020). In South American killifish, such oscillations are known to induce upregulation of heat shock proteins expression (Podrabsky and Somero 2004), suggesting that also in wild *N. furzeri*, cyclical thermal shock likely induces stress-response pathways. Finally, activity levels also differ between wild and captive animals even though it is difficult to predict how they can impact gene expression.

Genes upregulated in wild fish were particularly enriched in clusters of terms that are related to two main functions: mitochondrial respiration and translation and cell cycle/DNA replication. Mitochondrial respiration is a key regulator of aging. Partial inhibition of mitochondrial respiration is life extending in nematode worms, fruit flies and *N. furzeri* (Dillin et al. 2002; Copeland et al. 2009; Baumgart et al. 2016). Nuclear genes coding for proteins of the respiratory chain are under positive selection in multiple clades associated with the evolution of exceptional lifespan (Sahm et al. 2019), expression of complex I components negatively correlates with lifespan in interspecific comparison of mammals (Mota-Martorell et al. 2020) and rate of production of reactive oxygen species (ROS) by the complex I of the respiratory chain is inversely correlated with lifespan (Munro et al. 2013). The transcriptional coordination of mitochondrial translation and synthesis of nuclearly encoded components of the respiratory complex is termed mitonuclear balance and is a key regulator of aging across metazoans (Houtkooper et al. 2013; Baumgart et al. 2016). Therefore, wild and captive fish differed in the expression of the key genetic pathway for the regulation of aging. These differences may be a consequence of the activation of stress-response pathways in wild fish. Differences in the expression of cell cycle and DNA replication genes are paradoxical because wild animals have ceased growing. One possible explanation is activation of DNA repair pathways. A second possibility may be related to the circadian oscillation in the activity of adult neuronal stem cells of teleosts (Akle et al. 2017). The diel temperature oscillation may entrain the circadian rhythm leading to higher daytime mitotic activity in wild animals.

Genes upregulated in captive animals were enriched for GO terms related to neuronal communication and synaptic function. This result is surprising since exposure to a more complex environment is expected to induce synaptogenesis (van Praag et al. 2000). However, several factors may explain this seemingly paradoxical effect. In the first place, the brain of captive animals grows considerably during the examined period while captive animals have reached asymptotic size. Second, the stress levels of wild animals may curb synaptogenesis.

When age-dependent regulation was compared between wild and captive animals, more than 85% of the significant cases showed a consistent direction of regulation indicating a high degree of conservation between aging in the wild and in captive conditions. Considering that wild fish did not grow between the time points sampled, this result shows that most gene expression changes are not related to age-dependent deceleration of growth observed in captive animals. Downregulated genes showed overrepresentation of a large number of GO terms (supplementary table S9, Supplementary Material online) with the most significant enrichment for regulation of the mitotic cell cycle, protein folding, and chromatin but also neuronal development and formation of neuronal connection. This signal is clearly related to a reduction of neurogenesis with the formation of new connections by newborn neurons. Upregulated genes showed overrepresentation of a large number of GO categories (supplementary table S9, Supplementary Material online) with the most significant enrichment for terms related to translation, response to oxidative stress, and autophagy. The relationship of these processes with aging is well established (López-Otín et al. 2013; Steffen and Dillin 2016). Of interest is the upregulation of the Polycomb repressive complex, as its activity increases with age in *N. furzeri* (Baumgart et al. 2014; Cencioni et al. 2019) and sites targeted by Polycomb show age-dependent methylation in mammals (e.g., Minteer et al. 2022). Genes with opposite regulations also showed overrepresentation of specific GO terms and provide some interesting insights. Genes upregulated in captivity but downregulated in the wild show the most significant enrichment for genes coding for synaptic proteins. It should be noted that these GO terms also show significant differences in baseline conditions. This result suggests that brain connections develop differently in captive and wild environments. Genes upregulated in wild and downregulated in captivity show the most significant enrichment for DNA replication. This result is surprising and shows a disjunction in the regulation of cell-cycle-related genes and may be related to the heterochrony of somatic growth between wild and captive fish. Captive fish show continuous deceleration of growth during adult life, and this is reflected in a reduction of mitotic activity of adult neuronal precursors (Tozzini et al. 2012). Wild fish of the population examined, on the other hand, completed their growth during the first 5 weeks and could show the effects of aging isolated from somatic growth. The heterochrony of ontogeny and the more rapid development of wild fish are also reflected in the time-dependent age expression assessed by using monotonous genes as biomarkers. When captive-fish biomarkers were assessed in wild fish, their curves were left-shifted indicating that comparable expression levels were reached earlier in the wild. Wild-fish biomarkers, on the other hand, reflected genes whose expression was age-dependent in the absence of somatic growth. In this case, both starting values and slopes differed. The two combined observations indicate more rapid transcriptomic aging in wild fish. Interestingly, slower demographic aging rates were recently demonstrated for captive populations of turtles when compared with wild turtles (da Silva et al. 2022), indicating that other vertebrate taxa also respond to more favorable conditions by reducing the rate of senescence.

A particularly interesting result identified the contrasts in the DNA repair/DNA recombination pathway. This pathway shows age-dependent upregulation in wild fish but is downregulated with age in captive fish. The robust implication of this pathway in the evolution of longevity originates from several independent lines of evidence. Positive selection in genes coding for proteins related to DNA repair and homologous recombination was associated with evolution longevity in rockfishes (Kolora et al. 2021), cetaceans (Tollis et al. 2019), and long-living Galapagos tortoise (Quesada et al. 2019). Most relevant for the present study is a recent comparison of 45 killifishes of varying life history detected pervasive relaxation of positive selection on genes coding for DNA repair proteins in annual (short-lived) species (Cui et al. 2020). Complementary evidence to sequence analysis is provided by studies of expression profiling. One study correlated gene expression levels with longevity in a phylogenetically broad collection of 33 species of terrestrial mammals and identified DNA repair pathways to be overrepresented among genes whose expression is positively correlated with size-corrected lifespan (Fushan et al. 2015). This result was replicated and extended in a larger study focused on 6 tissues of 26 species of Rodentia and Eulipotyphla (Lu et al. 2022), and high expression of DNA repair genes was detected also in the gray whale (Toren et al. 2020).

At the single gene level, genes coding for proteins of the double-strand repair pathway were originally for their ability to complement DNA damage induced by X-ray (XRCCs). The gene XRCC1 is under positive selection in rockfishes (Kolora et al. 2021), the gene XRCC6 is under positive selection in the giant tortoise (Quesada et al. 2019), and expression of the genes XRCC5 and XRCC6 is positively correlated with lifespan in two mammalian datasets (Fushan et al. 2015; Lu et al. 2022). These genes have opposite regulation in captive and wild *N. furzeri*, and we confirmed the opposite age-dependent regulation of XRCC2 also by qPCR.

At a functional level, the activity of double-strand repair pathways declines with age in mice (Vaidya et al. 2014). On the other hand, long-living mammals show the higher basal activity of the double strand break (DSB) repair pathway (Tian et al. 2019). Interestingly, a longitudinal study of gene expression in *N. furzeri* revealed that the slope of age-dependent downregulation in the expression of genes in the DNA repair pathway is associated with a shorter lifespan (Kelmer Sacramento et al. 2020). On the other hand, longitudinal studies in long-living bats revealed age-dependent upregulation of the same pathway (Huang et al. 2019). It is notable that the age-dependent regulation of this pathway is different between wild and captive fish, suggesting that the wild conditions entail stimuli able to activate this pathway.

From the evolutionary perspective, gene regulatory mechanisms were conserved between wild and captive fish, suggesting the existence of conserved regulatory trajectories of gene expression employed across environmental conditions. Those trajectories were modified by developmental dynamics. Wild fish displayed rapid grow and reached growth asymptote before the first sampling. Irrespective of growth cessation, their temporal changes in gene expression were then in captive fish, and we propose that wild fish displayed accelerated aging. The pace of aging is known to respond to environmental conditions and contrasting populations of single species often display population-characteristic aging patterns (marsupials: [Austad 1993], snakes: [Bronikowski 2008], invertebrates: [Tatar et al. 1997; Dudycha and Tessier 1999], guppy: [Reznick et al. 2004], killifish: [Terzibasi et al. 2008; Blazek et al. 2017]). To recognize how mutation load (Willemsen et al. 2020) or epigenetic modification of gene expression affects this variation in gene expression dynamic is important for understanding the evolution of aging at a microevolutionary scale, and annual killifish appear ideally suited to contribute to resolving this question.

In conclusion, our study reveals that, despite baseline levels of brain gene expression varied between samples from wild and captive populations, the direction of regulation is mostly consistent. Intersexual differences in brain gene expression were minimal and in part related to sex determination. However, some pathways were differentially regulated in the two conditions, notably pathways related to DNA repair mechanisms, and this could result from heterochrony in the ontogeny.

## Experimental Procedures

### Sample Collection

Wild fish were collected by seine nets in natural ponds and immediately dissected. The collection and dissection took place between 7:00 and 9:00 h, that is, 1–3 h after sunrise. Upon dissection, the brain and other organs were stored in RNAlater at 4 °C and frozen at −80 °C after arrival in the laboratory (within 1–2 weeks after collection). Fish for dissections were chosen randomly from a set of captured fish. Their body size was measured. The age of the fish was estimated from the time of pool flooding and was confirmed by reading the number of daily rings on otoliths (Vrtilek, Zak, Polacik et al. 2018). Tissue from captive fish was collected as described (Baumgart et al. 2014).

### Fish Husbandry and Sampling

Fish were housed in a recirculating system (Aqua Schwartz, GmbH, Göttingen, Germany) and were singly housed in 2.8 l tanks with a divider. Water temperature was maintained at 26 °C and conductivity at 600 µS/cm. Animals were subject to a 12:12 light/dark cycle and fed exclusively with *Chironomus* larvae.

Animals were always sacrificed fasted in the morning, and the entire brain was extracted and immediately frozen on dry ice.

### Ethical Statements

All work with animals was carried out in accordance with relevant guidelines and regulations. Sample collection of wild animals complied with legal regulations of Mozambique (collection licence: ADNAP-170/7.10/16) and research procedures were approved by the ethical committee of the Institute of Vertebrate Biology, in accordance with legal regulations of the Czech Republic.

Captive fish were bred and kept in FLI’s fish facility under licence number J-003798 (Veterinär- und Lebensmittelüberwachungsamt, Thuringia, Germany). Sacrifice and organ harvesting were performed according to §4(3) of the German Animal Welfare Act. No experimental procedure on live animals was carried out.

### RNA Extraction

RNA was extracted as described (Baumgart et al. 2014) with a modified Trizol protocol, using Qiazol lysis reagent (Qiagen). In brief, samples were homogenized and lysed in QIAzol. After 5 min at room temperature, Chloroform was added and samples were mixed. After 3 min at room temperature, samples were centrifuged at 4 °C in a tabletop centrifuge. After phase separation, the upper aqueous phase was withdrawn and mixed with 1.1 volumes of isopropyl alcohol, 0.16 volumes 2M, pH 4.2 Sodium acetate, and 1 µl (10 µg) of GlycoBlue. Samples were precipitated by centrifugation for 30 min at 4 °C, washed twice with 80% ethanol, and air-dried for 5 min. RNA was dissolved in nuclease-free water.

### RNA Sequencing

Sequencing of RNA samples was performed using Illumina’s next-generation sequencing methodology. In detail, total RNA was quantified, and quality-checked using Agilent 2100 Bioanalyzer Instrument (Agilent RNA 6000 Nano). Libraries were prepared from 500 ng of input material (total RNA): GSE183039—TruSeq Stranded mRNA kit (Illumina) following the manufacturer’s instructions with subsequent quantification and quality check, using Agilent 2100 Bioanalyzer Instrument (DNA 7500 kit). Libraries were pooled and sequenced in 4 lanes of the HiSeq 2500 System running in 51 cycle/single-end/high output mode. Sequence information was converted to FASTQ format using bcl2fastq v1.8.4.
GSE183037—NEBNext Ultra II Directional RNA Library Prep Kit was prepared in combination with a NEBNext Poly(A) mRNA Magnetic Isolation Module (both New England Biolabs) following the manufacturer’s instructions. Libraries were subsequently quantified and quality-checked using Agilent 2100 Bioanalyzer Instrument (DNA 7500 kit). Libraries were pooled and sequenced in 2 lanes of the HiSeq 2500 System running in 51 cycle/single-end/high output mode. Sequence information was converted to FASTQ format using bcl2fastq v2.20.0.422.

Per sample, the reads were mapped to the *N. furzeri* genome (NFINgb—*N. furzeri* Genome Browser: https://nfingb.leibniz-fli.de/data/raw/notho4/Nfu_20150522.hardmasked_genome.fa.gz) and respective annotation (NFINgb: https://nfingb.leibniz-fli.de/data/raw/notho4/Nfu_20150522.genes_20150922.gff3.gz) using tophat2 v2.1 (parameters: –no-convert-bam –no-coverage-search -x 1 -g 1). Reads per gene were counted using featureCounts v1.5 (GSE183039) or v1.6.5 (GSE183037) (parameters: -s 2). Reads counts were introduced into the statistical environment R in order to calculate (reads per million mappable reads) and RPKMs (reads per kilobase and million mappable reads). For the calculation of RPKMs, lengths of transcripts were taken from featureCounts output.

The statistics relative to the different samples are reported in supplementary table S12, Supplementary Material online.

### Variant Analysis

Variant calling was performed on the aligned BAM files using bcftools (Li 2011) and then used for genotype calling and identity-by-descent (IBD) analysis using the SNPRelate package (Zheng et al. 2012). For the first analysis, linkage equilibrium through LD-based SNP pruning was applied to the variants, and then genotypes for each SNP were obtained. For the second one, kinship values among the samples were measured using the Maximum Likelihood Estimation method.

### Differential Analysis

To eliminate low-expressed genes, we filtered out all genes whose raw expression was not ≥1 count in all samples, excluding 5,220 genes out of the 23,545 protein-coding genes annotated in the *N. furzeri* genome. Differentially expressed transcripts in wild animals were obtained with the DESeq2() package (Love et al. 2014). To compare wild and captive animals, we used the MACAU software (Sun et al. 2017) to correct genetic relatedness among captive animals. MACAU models one gene at a time and assumes that the reads are distributed according to a Poisson distribution. If *N_i_* is the total number of reads in a sample and *λ_i_*is the fraction of *N_i_* mapping to the gene of interest, the distribution of reads is defined by:

$$y_{i}=Po_{i}(N_{i}\lambda_{i})$$

MACAU further assumes that *λ_i_* can be modeled as follows:

$$\log(\lambda_{i})=w_{i}\alpha +x_{i}\beta +g_{i}+e_{i}\;$$

where *w_i_ α i*s the linear combination of covariates, *x_i_* is the predictor variable (age or wild/captive in our case), and *β* is its weight. The last two terms model the contribution of genetic relatedness and the error term.

MACAU further assumes that both terms are defined by a multivariate normal distribution, in the case of *g*; this contribution is defined by the covariance matrix *K* (i.e., a normalized IBD matrix) and the heritability of gene expression *h* that we set to 0.4.

$$\begin{array}{c} \bar{g}=(g_{1},\ldots ,g_{n})∼MVN(0,\sigma^{2}h^{2}K)\\ e=(e_{1},\ldots ,e_{n})∼MVN(0,\sigma^{2}{\left(1-h\right)}^{2}I_{n\times n}) \end{array}$$

where *I_n × n_*is the identity matrix of dimension *n*.

We have used the MACAU analysis twice. In the first analysis, we set age as covariate and condition as a predictor variable to identify genes differentially expressed in wild animals. In the second analysis, we analyzed the effects of age as a predictor variable on the two conditions separately and then plotted, for each DEG, *β*_wild_ versus *β*_captive_.

### Enrichment Analysis

Gene ontology was performed for each tissue with the WebGestalt online tool using GO process; enriched categories for each quadrant were filtered for FDR < 0.05.

Generally Applicable Gene-set/pathway enrichment (GAGE) was performed using the gage() package; enriched categories were filtered for *q*-value < 0.05 and used for visualization with the Revigo software. Entrez IDs were used as identifiers for the analysis, from the human orthologues gene symbols: these were obtained with the biomart() package.

### Principal Component Analysis

Visualization of the data (for both transcriptome) was performed with PCA: DEGs for different feature selection were obtained and selected for the analysis. Principal components of the samples were obtained through the prcomp() function and then plotted, after computing centroids as means of the replicates for each group of samples.

### Aging Biomarkers Isolation

Aging biomarkers were computed as genes with linear expression trajectories during time: sequential pairwise comparisons across time points were obtained with the DESeq2() package (Love et al. 2014) and used to filter genes with fold-changes >0 (for positive biomarkers) or <0 (for negative biomarkers) in all comparisons. The *P*-values of all pairwise comparisons were then combined using meta-analysis, and the final *P*-value was adjusted to FDR using the p.adjust() function. Significant biomarkers were isolated for FDR < 0.05. To plot the expression trajectories over time in captive and wild animals, count data were cleaned for technical covariates (specifically for RIN coefficient) using the cleaningY() function from the Jaffelab GitHub repository (Collado-Torres et al. 2017), then were converted to z-score (centering each value on the mean and scaling on the standard deviation), and finally used to plot trajectories using the ggplot2 package (Wickham 2016).

### Experimental Validation

Experimental validation was done by RT-qPCR from wild and captive RNA, using the primers listed in supplementary table S11, Supplementary Material online. In brief, 500 ng per sample was reverse transcribed with SuperScript IV Reverse Transcriptase (Invitrogen). Quantification was performed by use of Quantinova SYBR Green (Qiagen) with CFX384 real-time PCR system (Biorad). For the validation of condition-dependent genes, we used a total of 16 samples (8 wild at 39 phd and 8 captive at 5 wph), using TATA Binding Proteinn (TBP) for normalization. For the validation of age-dependent genes, we used a total of 16 samples, comprising 8 wild (4 at 39 dph, and 4 at 108 dph), and 8 captive fish (4 at 5 wph and 4 at 12 wph). For the visualization, we used each point, which represents a replicate, whereas the middle segment represents the median of the distribution. A *T*-test was used for statistics.

## Supplementary Material

msac219_Supplementary_Data

## Data Deposition

Data from this study were deposited in gene expression omnibus, accession numbers GSE183037, GSE183039 and GSE52462.

***Conflict* *of interest statement***. The authors declare no competing financial interests.
