# Supplementary material for: RNAseq Analysis of Brain Aging in Wild Specimens of Short-Lived Turquoise Killifish: Commonalities and Differences With Aging Under Laboratory Conditions
Source: Mol Biol Evol. 2022 Nov 1;39(11):msac219. doi: 10.1093/molbev/msac219 (PMC9641980; doi:10.1093/molbev/msac219)
Supplement: msac219_Supplementary_Data [file msac219_supplementary_data.zip › FigS1.pdf]

Fig. S1

Wild fish pop. A41

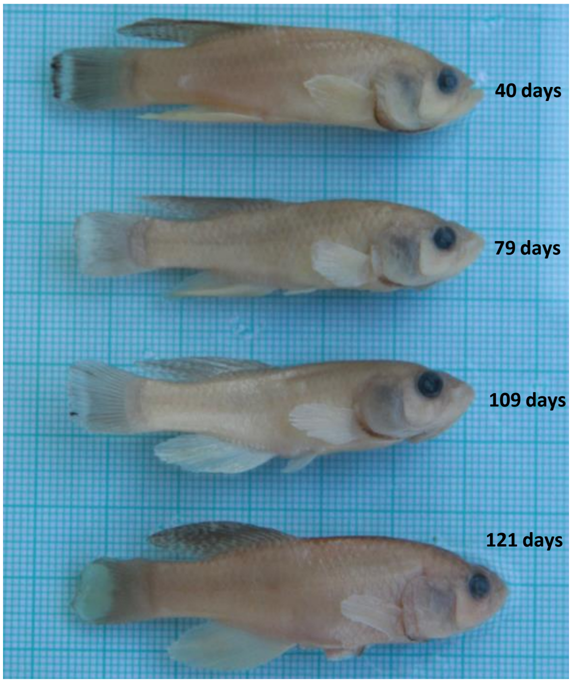

Captive fish, strain MZM04010

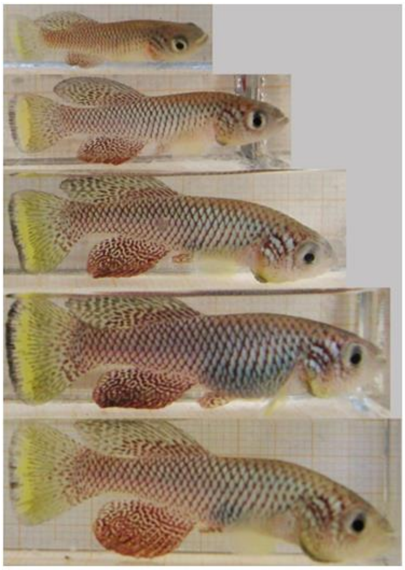

35 days

77 days

133 days

203 days

273 days

Representative series of wild and captive males with the corresponding ages. Note the lack of somatic growth for wild fish during the illustrated period
